# Friedenheim Hospital

**Published:** 1899-06-17

**Authors:** 


### FRIEDENHEIM HOSPITAL.

The council and supporters of Friedenheim, the Home of Peace for the Dying, held their annual meeting in the adjoining lecture hall for the blind on Thursday, the 8th inst. The meeting was well attended, and among those on the platform were the Rev. Canon Girdlestone, M.A., Dr. S. H. Habershon, F.R.C.P., Mr. Algernon C. P. Coote, and Dr. A. F. Schofield, M.R.C.S. Mr. J. H. Fritton (hon. treasurer) presided. The medical report stated that nearly four-fifths of the total number of cases received were of consumption and cancer.

The Chairman, in the course of a brief opening speech, said the addition of hospital to the name of the institution had been judged advisable. Friedenheim, *i.e.*, home of peace, in some cases required to be translated before its object and meaning were recognised.

Dr. Habershon, in addressing the meeting, referred to the noble work Miss Davidson, the hon. superintendent, had done in founding the home. The original home, which contained only ten beds, was started in 1885, and seven years later the present establishment was opened at Swiss Cottage. Friedenheim, he said, was not intended for the benefit of the indigent poor, to whom the infirmaries were open, but for the superior working-class and people in reduced circumstances. In this home of peace the gloomy side of death was kept in the background, the surroundings of the patients were cheerful and bright, and they had not only bodily tendance, but were helped to a hopeful religious outlook. Referring to the great need of improved accommodation for the nurses, Dr. Habershon said the rooms they at present occupied were over a stable which had fallen out of repair. As the cost of pulling down and rebuilding the stable would be almost as great as that involved in erecting the proposed new nurses' quarters, in undertaking the building of the latter the council could not be said to have outrun economy.

Mr. Algernon Coote was the next speaker. He stated that there were 42 beds in Friedenheim, the average cost of each being about 10s. per week. The work was carried on as a labour of love, which accounted for the comparatively small cost of administration. As, since the beginning of the year, the ordinary income had not sufficed for the needs of the home, the Council had been compelled to draw upon the £500 which formed the nucleus of the building fund. Therefore, rather more than £2,500 was asked for to complete the £3,000, which represented the total cost of erecting the new nurses' home. A very great strain, the speaker said, was involved in constant attendance on the dying. An occasional complete change of surroundings and atmosphere would be immensely beneficial to the nurses' health and add to the value of their services. Their present accommodation was situated in the main building.

Canon Girdlestone, in an earnest speech, alluded to what to him seemed the amount of unnecessary labour, chiefly mechanical, that fell to women in hospitals that could not but render them less fit for their real work of nursing. There was, in general, he observed, a noticeable want of thought and care for the nurses in hospitals. In order to impart to the patient what is so important—a spirit of hopefulness—the nurse herself must be feeling fresh and hopeful.

A vote of thanks to the chairman concluded the meeting.

